# Interdisciplinary training program for pediatric cardiorespiratory arrest using rapid cycle deliberate practice: A descriptive cross-sectional study

**DOI:** 10.1590/1516-3180.2023.0271.16022024

**Published:** 2024-06-17

**Authors:** Renata Pereira, Edina Mariko Koga da Silva

**Affiliations:** I Master’s student; Department of Medicine; Universidade Federal de São Paulo (UNIFESP). São Paulo (SP), Brazil; II Associate Professor; Department of Medicine; Universidade Federal de São Paulo (UNIFESP). São Paulo (SP), Brazil

**Keywords:** Simulation training, Interprofessional education, Cardiopulmonary resuscitation, Continuing education, Rapid-cycle deliberate practice, Simulation, Pediatric resuscitation, Learner perception, Heart Attack

## Abstract

**BACKGROUND:**

cardiorespiratory arrest (CRA) is a severe public health concern, and clinical simulation has proven to be a beneficial educational strategy for training on this topic.

**OBJECTIVE:**

To describe the implementation of a program for pediatric cardiac arrest care using rapid-cycle deliberate practice (RCDP), the quality of the technique employed, and participants' opinions on the methodology.

**DESIGN AND SETTING:**

This descriptive cross-sectional study of pre- and post-performance training in cardiopul monary resuscitation (CPR) techniques and reaction evaluation was conducted in a hospital in São Paulo.

**METHODS:**

Multidisciplinary groups performed pediatric resuscitation in a simulated scenario with RCDP mediated by a facilitator. The study sample included professionals working in patient care. During the simulation, the participants were evaluated for their compliance with the CRA care algorithm. Further, their execution of chest compressions was assessed pre- and post-intervention.

**RESULTS:**

In total, 302 professionals were trained in this study. The overall quality of CPR measured pre-intervention was inadequate, and only 26% had adequate technique proficiency, whereas it was 91% (P < 0.01) post-intervention. Of the participants, 95.7% responded to the final evaluation and provided positive comments on the method and their satisfaction with the novel simulation. Of these, 88% considered that repetition of the technique used was more effective than traditional simulation.

**CONCLUSIONS:**

The RCDP is effective for training multidisciplinary teams in pediatric CPR, with an emphasis on the quality of chest compressions. However, further studies are necessary to explore whether this trend translates to differential performances in practical settings.

## INTRODUCTION

Cardiorespiratory arrest (CRA) is considered among the most severe public health problems due to its significant healthcare burden, with significantly high morbidity and mortality rates.^1–3^

In pediatric patients, this condition is infrequent, and when it occurs, it is usually the result of respiratory failure progression. For this reason, asystole and pulseless electrical activity (PEA) are the most frequently presented rhythms, as evidenced in a study by Skellett et al. in the United Kingdom.^4–6^ In this scenario, defibrillation is not recommended, and high-quality cardiopulmonary resuscitation (CPR) is the most preferred intervention in this population.^4,7^

Each year, approximately 19,900 pediatric cardiac arrests occur in the United States, according to the United States of America CRA registry publication.^3,8,9^

In Brazil, the database analysis of a tertiary pediatric hospital emphasized that in 15 years, an increase of 6% was observed in the reestablishment rate of spontaneous circulation and that of 13.8% in survival at hospital discharge.^9,10^ This improvement is likely due to multifactorial reasons that can be attributed to earlier recognition and management of at-risk patients, improved quality of basic and advanced life support, post-CRA care, and patient care team training.^3^

Despite evidence supporting that CPR (cardiopulmonary resuscitation) is the best practice in CRA treatment, the technique quality provided to pediatric patients in simulated and real CRA situations^11,12^ is often inadequate and may negatively impact survival rates.^13–15^

Therefore, effective CPR training requires the use of innovative educational strategies to achieve optimal results,^14^ and clinical simulation has proven to be a beneficial educational strategy. It supports skill development by reproducing a desired clinical context in a safe and controlled environment.^16^

Traditionally, clinical simulation consists of complete scenario development by a group of participants, followed by debriefing mediated by a facilitator.^17^ After which the scenario is terminated without the participants having the opportunity to practice the required tasks again in the correct manner.^17,18^

To solve this problem, a study by Anders Ericsson^19^ suggested deliberate practice as the most effective method to achieve perfection in performance. This method aims to maximize performance through well-defined learning objectives, focused and repetitive practice, accurate performance measures, and informative feedback.^19,20^

Given these conditions, the use of the rapid-cycle deliberate practice (RCDP), a simulation strategy created by Hunt, has been recommended.^21^ This is a simulation-based model of education in which learners develop their actions repeatedly (deliberate practice) for a short period (rapid cycle) in the same clinical scenario until the desired skill is mastered by the participant. When the objectives of one cycle are achieved, a new cycle begins, increasing the complexity of the tasks required.^18,20^

The RCDP described by Hunt is characterized by a combination of personalized directive feedback and practice, which is repeated according to the participating students' performance. It modifies the debriefing from traditional post-simulation feedback to directive feedback within an event.^22,18^ In this novel approach, whenever there is a non-conformity, the scenario is paused, the students are interrupted, and the instructor provides brief, pertinent corrective information.^21–23^ After the corrections, the scenario continues, but this time follows the correct process. It allows participants to have multiple opportunities to perform the technique correctly by receiving focused guidance until they master the desired skills.^24,25^

## OBJECTIVE

The main objective of this study was to describe the implementation of a simulation-based interdisciplinary training program using RCDP specific to the context of CRA in pediatrics. Other objectives were to describe the quality of performance of the professionals at the beginning and end of the training and to report the participants' opinions regarding the methodology used.

## METHODS

This descriptive cross-sectional study was conducted in a private hospital providing pediatric care exclusively in the city of São Paulo from November 2020 to May 2022.

For this training project, the focus was a pediatric CRA with a high-performance team. For this purpose, a fully simulation-based multidisciplinary small group activity was established and supported by the most updated methodology and evidence in the field of medicine.

The convenience sample comprised 302 professionals including nurses, physical therapists, and physicians who work at the institution, regardless of hierarchical level and area of work, who agreed to participate in the study and signed an informed consent form. Those who did not participate in the training or sign the informed consent form were excluded.

The participants were invited to participate in the study and informed of its objectives. Participation was voluntary, and confidentiality was maintained.

After they consented to participate, they received an online form to fill in the demographic data, a brief instruction with a review of the Pediatric Advanced Life Suport (PALS-2020)^26^ algorithm, guidance on the methodology to be used in training, and its objectives. They then began the care of a simulation case using a high-fidelity manikin sequenced in cycles. With each cycle, additional steps of the pediatric resuscitation algorithm were added.

The performance of the participants selected for chest compressions was evaluated in the first cycle of care (without instructor intervention) for frequency, depth, and quality of technique using the RescueNet Code Review software (standard edition) from Zoll Medical Corporation (Chelmsford, Massachusetts, USA), which captures information from electrodes positioned on the manikin and archives it for later analysis.

The parameters configured in the software as the quality standard for the technique were based on the guidelines determined by the American Heart Association (AHA).^26^ For the variable compression frequency, the range of 100–120 compressions/min was defined, for depth compressions with 4–5 cm anteroposterior chest diameter. To determine the quality of the technique according to this guideline, parameters such as frequency and depth must occur simultaneously.

During the simulation, participants were evaluated for compliance with the CRA care algorithm. Therefore, when the instructor noticed a failure to reach a goal for that round, the scenario was paused and feedback was provided to correct the error, after which the activity was restarted from the beginning of that round, allowing the participants to practice the skills correctly.

The scenario was completed once the team achieved its goals for all cycles. Finally, in the goal-achieved cycle, the participants' performance in chest compressions was also recorded, collecting data following the same parameters as those in the first cycle.

At the end of the training, a reaction survey was made available to the participants to evaluate the training without identifying the respondents. The form consisted of basic demographic information, multiple-choice questions using a Likert scale to collect information on perceived practicality and possible points for improvement, basic information on the participants, and a space for comments.

Data from the reaction evaluation questionnaire and the feedback software were statistically analyzed. The average and standard deviation were calculated for the continuous variable of CPR quality with a t-test comparison of the average recorded pre- and post-training.

This study was approved by the Ethics Committee of Universidade Federal de São Paulo (approval number: 4,195,837) on August 6, 2020.^27^ Its results will only be disclosed anonymously, and it will not be possible to identify its participants in compliance with the General Data Protection Law.^28^

## RESULTS

For simulation, a pediatric CRA case was adapted for use in the RCDP methodology. Cycles were created to provide the participants with adequate repetition of established goals and new challenges at each stage. This case was then used to train instructors.

During training, each instructor had the opportunity to lead a round of simulations, while the others enacted the clinical case. In addition, all trainers who participated in the additional training focused on deliberate practice methodology.

The safe learning environment generated integration and engagement from the instructors, and important contributions were made to enhance the performance of the simulated scenario. Moreover, they clarified doubts, defined expected standards for the participants, discussed and aligned the proposed learning objectives, and practiced the adopted methodology.

This qualification was an important part of the project as it enabled standardized training for all instructors. In this process, the challenge was to transition from the traditional simulation methodology, already well-established by previous training, to RCDP.

**Figure 1:**
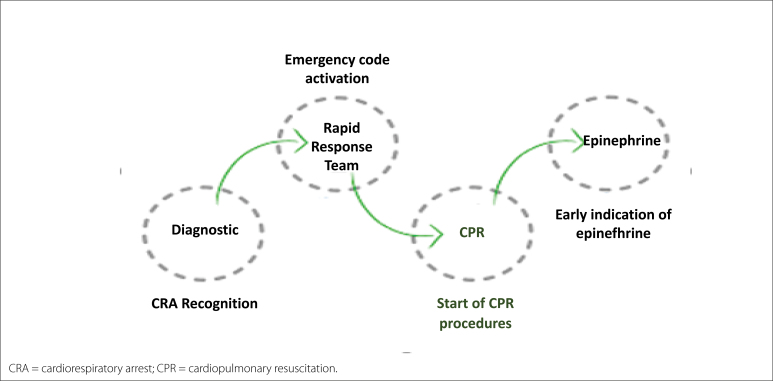
Goals set for each training session.

The clinical case scenario corresponded to a CRA in the PEA rhythm resulting from severe respiratory failure and had four objective goals (Figure 1).

Each program round was conducted by two instructors: one responsible for operating the scenario and the other for recording and extracting performance data from the equipment.

**Figure 2:**
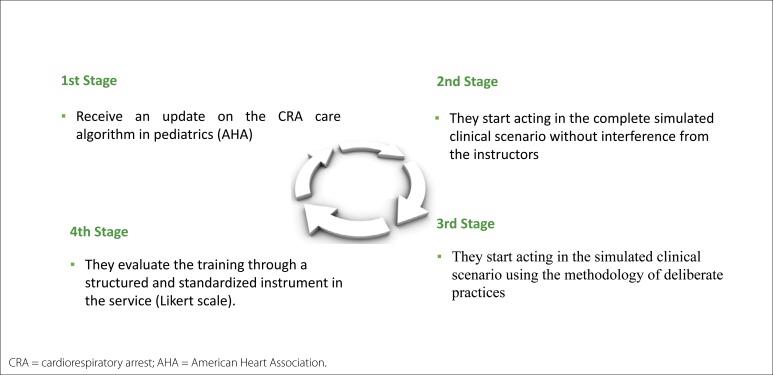
Steps established for each training session – rapid-cycle deliberate practice.

The group sessions were 1 h long and divided into 50 min of instructional time and 10 min of course feedback and evaluation (Figure 2).

The sessions were conducted from November 2020 to May 2022, and each session had six participants, as determined by the institution's CRA care protocol. The data resulting from the maneuvers of the two professionals who took turns performing chest compressions were recorded in the defibrillator equipment software in the first and last cycles of training and used later for the analysis.

The 50 min of instruction time was divided as follows: the first 10 min for presenting the objectives, program strategy, and a punctual review of the CRA care algorithm determined by the AHA in 2020. The next 10 min were for observation of the care provided by the team in the CRA scenario without interruptions or interventions by the instructors (recording of pre-intervention domain training data). Finally, the remaining 30 min were allocated to the same scenario but this time with the RCDP methodology applied. The progress of the service was paused when an action was incorrect, feedback was directed at failure, and the team was provided another opportunity to correctly perform the technique. Post-intervention compression data were also recorded during the last cycle of care in this scenario.

Finally, 10 min were reserved for closing feedback, in which each group was presented with a graph of the quality of chest compressions before and after the training so that they could visualize the difference in performance. Further, a course feedback evaluation using a structured online form was made available in the end.

Owing to the Sars-CoV-2 pandemic, the institutional norms related to infection control were adjusted, and the training sessions were linked to their compliance to ensure the safety of participants and instructors.

These new guidelines directly impacted the planning of this project. To comply with these guidelines, the number of professionals in the training area was limited, and the location was changed to an external area of the hospital (outdoors), adapted, and prepared with pediatric emergency equipment.

In addition, the attendees wore a mandatory N95 mask during the entire training period, hand sanitization with alcohol gel usage was imposed at the beginning and end of each session, and the equipment and materials used were sanitized with standardized hospital disinfectants at the end of each group.

All support materials and training forms were made available electronically so that participants could access them from their cell phones and, as such, did not share materials.

The entire project was studied and evaluated to ensure that safety protocols were strictly adhered to, guaranteeing safety for participants and instructors while not compromising training dynamics.

**Graph 1:**
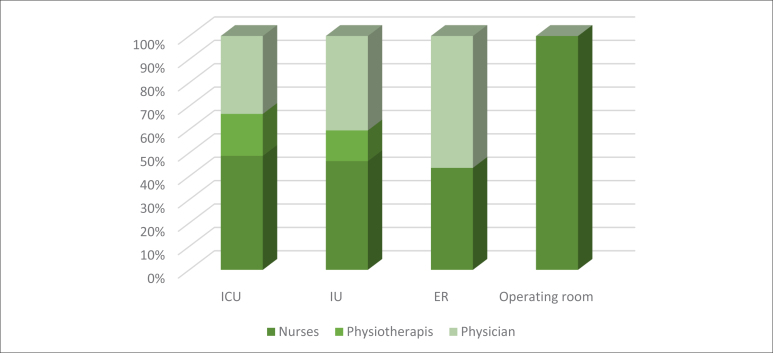
Distribution of CPR training participants with RCDP by activity unit; nov 2020 to mar 2022. (n = 302)––

Overall, 302 professionals were trained during the study period, and all records of the participants' performance in chest compressions and responses to the reaction evaluation form were included in the analysis. The sample comprised 48% nurses, 41% physicians, and 11% physical therapists. Most participants performed their care activities in the intensive care unit, followed by inpatient units, emergency rooms, and operating rooms (Graph 1).

Among the training participants, 89% had already participated in a course on CRA care in pediatrics (Table 1).

**Table 1:** Participation in a previous pediatric CRA course

	N	Yes N(%)	No N(%)
Nurses	145	128 (88%)	17 (12%)
Physiotherapists	34	24 (71%)	10 (29%)
Physician	123	119 (97%)	4 (3%)

The overall quality of chest compressions measured at the pre-intervention educational mastery training time was inadequate, and only approximately 26% had technique quality within the given standards. In the post-intervention analysis, 91%, on average, reported the quality of the technique used according to the guidelines (Table 2).

**Table 2:** Performance of professionals in the CPR technique

compressions	N	AHA guideline	Pre-training average	DP	Pos-training average	DP
**Frequency**	302	100–120	109,62	14,36	109,59	5,89
**Depth**	302	4–5	4,41	0,83	4,21	0,86
**Quality of CPR**	**AHA guideline**		**Pre-training**		**Pos-training**	
	100%		26%		91%	

In the statistical analysis comparing the mean quality of the pre- and post-training proficiency using the t-test for paired samples, the difference was significant (P < 0.01).

**Graph 2:**
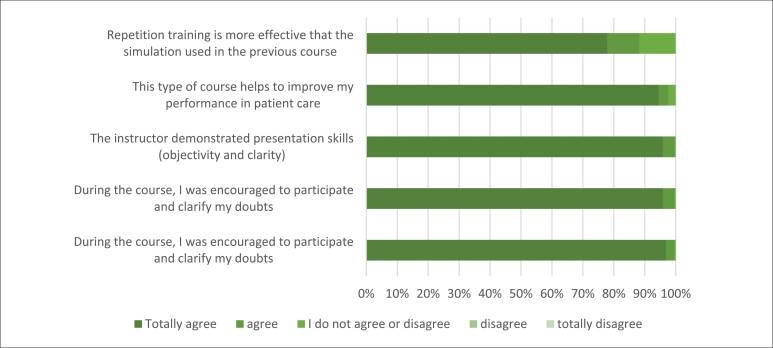
Reaction evaluation of CPR training participants with PDCR; nov 2020 to mar 2022. (n = 289)

Regarding the evaluation of the training, 289 answers were obtained from the form made available; that is, 95.7% of the participants registered their impressions of the training (Graph 2).

We observed that 97% of the respondents agreed that the theme was important for the practice of their daily assistance activities, and 95% agreed that the activity contributed to their performance improvement in direct patient care.

Analyzing the instructors' performance directly, 96% agreed that they were encouraged to participate and ask questions during the training and that the instructors were objective and clear in their teaching.

Regarding the method instituted for skill mastery training, 78% agreed that repetition of the technique used in this methodology was more effective than the traditional simulation of previous pieces of training.

## DISCUSSION

A team without appropriate knowledge of how to act in a crisis presents difficulties in the consistent execution of the guidelines that form the basis of CRA care. Therefore, professional education is important for improving survival outcomes in these cases.

This educational opportunity took advantage of the RCDP, which is best employed in simulations that require a participant to follow a clearly defined script under time pressure, as is the case in resuscitation scenarios. Therefore, the adopted methodology met the for skill requirements associated with the learning objectives defined for this project.

Among the training participants, 89% had attended a course on pediatric CRA on another occasion. Even with this prior theoretical knowledge and the review of current guidelines conducted immediately before training, the average performance measured in the pre-intervention scenario presented an inadequate quality of the chest compression technique.

From these findings, and consistent with other publications, it is possible to conclude that although theoretical presentations have a clear impact on learning that can be evidenced by knowledge assessment, they are not enough to ensure the transfer of knowledge acquired into practice during simulated clinical scenarios.^29,30^

The information extracted from the software showed that the average frequency and depth of the chest compressions executed before the intervention, despite being within the ranges determined by the AHA protocols, were of inadequate quality. Therefore, in most resuscitation movements, these two variables are not present simultaneously in the pattern.

This condition noticeably improved performance measured after training. Recording the resuscitation technique employed in the last RCDP cycle revealed that the frequency and depth averages remained within the given standards, and the quality of the technique employed was recorded as adequate in approximately 91% of the sessions.

The analysis comparing the average pre- and post-training proficiency using the paired samples t-test revealed that the training intervention resulted in significant improvement in technique quality (P < 0.01). This methodology can improve CPR skill quality.

This result aligns with the current literature, which shows immediate improvement in skills and, consequently, in individual and team performance when simulations using the RCDP technique are used in pediatric CPR.^31–34^

One of these studies was a pilot study by Lemke et al., which demonstrated a trend toward improved staff performance during pediatric CRA care after training with RCDP compared with traditional simulation. The study emphasized that the technique was well-received by students, but they reported fatigue due to repetition.^34^

Another study corresponding to our results is that of Hunt et al., who developed a teaching approach with RCDP that focused on the rapid acquisition of resuscitation and teamwork skills in the first 5 min of pediatric CRA care. The results revealed that training was associated with better resident performance during care for almost all measurable measures.^33^

Focusing on prehospital CRA care, Kosoko et al. developed training with RCDP and showed that this intervention was an effective way to educate professionals about this medical emergency.^35^

Regarding the evaluation of the training feedback, almost all respondents considered the topic covered in the training to be important for their practice. Among the placements in the free evaluation field, the most common one regards CRA as a severe situation, which is infrequent, and thus needs to be trained periodically to be performed with quality and safety.

These results also demonstrated that the training provided greater self-confidence in resuscitation skills. Participants perceived the proficiency they had achieved at the end of the sessions and were able to evaluate differences in technique performance after the RCDP cycles.

Consistent with the findings of this study, the mastery learning methodology is increasingly associated with better educational outcomes, especially concerning resuscitation topics,^20,31-34^ as highlighted by Magee et al.^32^

The survey items that evaluated the instructors emphasized the relevance of previous training and instructor involvement in scenario construction and clinical cases. These steps provided standardization in the process and commitment to the stipulated learning objectives.

In the last statement of the form, participants were encouraged to compare the traditional simulation performed earlier with the RCDP. The responses revealed that the participants perceived that their ability to practice repeatedly enhanced their learning regardless of how time-consuming it was.

Finally, the comments were almost entirely positive and allowed us to state that the dynamism of RCDP was well-received by the study participants. In line with the findings of Chancey et al.^36^

Regarding perceived effectiveness, most participants exhibited a high degree of satisfaction in recognizing the relevance of the content and skills learned. It is noteworthy that the present report is surprising at the results of the pre- and post-training comparisons. In fact, a large proportion realized and recorded that the techniques they believed they knew properly fell short of what was expected for a high-quality procedure.

Another important issue was the perception that it was productive to learn from other members of the multidisciplinary team because working in a cohesive team is a determining factor in successfully handling an emergency.

Although future studies are necessary to evaluate the translation of knowledge and skills from the learning environment to the clinical setting, this study revealed that it is critical to devote efforts to developing innovative health education strategies based on current scientific evidence for an improved care. These interventions can lead to differences in excellent and safe care for pediatric patients.

The results of this study reflect a specific simulated context, making it difficult to predict whether they can be directly generalized to actual patient care.

Another limitation is the lack of data on the improvement in CPR quality observed in previous training courses for comparison with the data obtained in this study.

Because it is a single-event training program, a higher limitation is the non-evaluation of skill retention. Therefore, it is impossible to assess how often training is required to maintain adequate professional performance.

## CONCLUSIONS

The results of this study suggest that the RCDP is an appropriate and effective methodology for interdisciplinary team training in pediatric CPR, with an emphasis on the quality of the chest compression technique.

The measurable data revealed a significant improvement in participants' performance in the last cycle of the scenario compared to the first cycle. This demonstrates that this method contributed to the improvement of adequate technical skills in compression during CPR care in a simulated environment.

However, further studies are needed to explore whether this presented trend translates into differential performance in a practical setting, whether these acquired skills are sustained in the long term, and for how long.

This program was effective, well-received, and well-evaluated by the participants, who considered it better, more practical, and more dynamic than the simulation methodology previously adopted as a standard.

Nevertheless, further research is needed to determine whether the perceived benefits of this mastery training program translate into clinical practice by reducing errors, improving team performance, and, most importantly, improving the prognosis of children who rely on high-quality CPR.

## References

[1] 1 Bimerew M, Wondmieneh A, Gedefaw G, et al. Survival of pediatric patients after cardiopulmonary resuscitation for in-hospital cardiac arrest: A systematic review and meta-analysis. Ital J Pediatr. 2021;47(1):118. PMID: 34051837; 10.1186/s13052-021-01058-9. PMC8164331 34051837

[2] 2 Bastarrica EG, dos Santos F, Conte M, Baldo APV. Epidemiological profile of cardiorespiratory parede patientes: An integrative review. RSD. 2020;9(12):e1559126024. 10.33448/rsd-v9i12.6024.

[3] 3 Hamzah M, Othman HF, Almasri M, Al-Subu A, Lutfi R. Survival outcomes of in-hospital cardiac arrest in pediatric patients in the USA. Eur J Pediatr. 2021;180(8):2513-20. PMID: 33899153; 10.1007/s00431-021-04082-3. 33899153

[4] 4 Meaney PA, Bobrow BJ, Mancini ME, et al. Cardiopulmonary resuscitation quality: improving cardiac resuscitation outcomes both inside and outside the hospital: a consensus statement from the American Heart Association. Circulation. 2013;128(4):417-35. PMID: 23801105; 10.1161/cir.0b013e31829d8654. 23801105

[5] 5 Skellett S, Orzechowska I, Thomas K, Fortune PM. The landscape of paediatric in-hospital cardiac arrest in the United Kingdom National Cardiac Arrest Audit. Resuscitation. 2020;155:165-71. PMID: 32768496; 10.1016/j.resuscitation.2020.07.026. 32768496

[6] 6 Mick NW, Williams RJ. Pediatric Cardiac Arrest Resuscitation. Emerg Med Clin North Am. 2020;38(4):819-39. PMID: 32981620; 10.1016/j.emc.2020.06.007. 32981620

[7] 7 Girotra S, Spertus JA, Li Y, et al. Survival trends in pediatric in-hospital cardiac arrests: an analysis from Get With the Guidelines-Resuscitation. Circ Cardiovasc Qual Outcomes. 2013;6(1):42-9. PMID: 23250980; 10.1161/circoutcomes.112.967968. PMC3555689 23250980

[8] 8 da Silva WM, Santos MP, Silva CAO, et al. Atualizações em parada cardiorrespiratória pediátrica: uma revisão. Int J Develop Res. 2021;11(5):47001-5. 10.37118/ijdr.21925.05.2021.

[9] 9 Shimoda-Sakano TM, Schvartsman C, Reis AG. Epidemiology of pediatric cardiopulmonary resuscitation. J Pediatr. 2020;96(4):409-21. PMID: 31580845; 10.1016/j.jped.2019.08.004. PMC9432320 31580845

[10] 10 Shimoda-Sakano TM, Paiva EF, Schvartsman C, Reis AG. Factors associated with survival and neurologic outcome after in-hospital cardiac arrest in children: A cohort study. Resusc Plus. 2023;13:100354; PMID: 36686327; 10.1016/j.resplu.2022.100354. PMC9852640 36686327

[11] 11 Cheng A, Hunt EA, Grant D, et al. Variability in quality of chest compressions provided during simulated cardiac arrest across nine pediatric institutions. Resuscitation. 2015;97:13-9. PMID: 26417701; 10.1016/j.resuscitation.2015.08.024. 26417701

[12] 12 Sutton RM, Niles D, French B, et al. First quantitative analysis of cardiopulmonary resuscitation quality during in-hospital cardiac arrests of young children. Resuscitation. 2014;85(1):70-4. PMID: 23994802; 10.1016/j.resuscitation.2013.08.014. PMC3877702 23994802

[13] 13 Lurie KG, Nemergut EC, Yannopoulos D, Sweeney M. The Physiology of Cardiopulmonary Resuscitation. Anesth Analg. 2016;122(3):767-83. PMID: 26562060; 10.1213/ane.0000000000000926. 26562060

[14] 14 Lin Y, Cheng A, Grant VJ, Currie GR, Hecker KG. Improving CPR quality with distributed practice and real-time feedback in pediatric healthcare providers: a randomized controlled trial. Resuscitation. 2018;130:6-12. PMID: 29944894; 10.1016/j.resuscitation.2018.06.025. 29944894

[15] 15 Idris AH, Guffey D, Aufderheide TP, et al. Relationship between chest compression rates and outcomes from cardiac arrest. Circulation. 2012;125(24):3004-12. PMID: 22623717; 10.1161/circulationaha.111.059535. PMC3388797 22623717

[16] 16 Gross IT, Abrahan DG, Kumar A, et al. Rapid cycle deliberate practice (RCDP) as a method to improve airway management skills–a randomized controlled simulation study. Cureus. 2019;11(9):e5546. PMID: 31523589; 10.7759/cureus.5546. PMC6721918 31523589

[17] 17 Castro LT, Couto TB. Prática Deliberada em Ciclos Rápidos: uma estratégia moderna de simulação. Sci Med. 2018;28(1):ID28849. 10.15448/1980-6108.2018.1.28849.

[18] 18 Taras J, Everett T. Rapid cycle deliberate practice in medical education - a systematic review. Cureus. 2017;9(4):e1180. PMID: 28540142; 10.7759/cureus.1180. PMC5441688 28540142

[19] 19 Anders Ericsson K. Deliberate practice and acquisition of expert performance: a general overview. Acad Emerg Med. 2008;15(11):988-94. PMID: 18778378; 10.1111/j.1553-2712.2008.00227.x. 18778378

[20] 20 Patricia K, Arnold J, Lemke DS. Rapid Cycle Deliberate Practice: Application to Neonatal Resuscitation. MedEdPORTAL Publications. 2017;13:10534. PMID: 30800736; 10.15766/mep_2374-8265.10534. PMC6342166 30800736

[21] 21 Hunt EA, Vera K, Diener-West M, et al. Delays and errors in cardiopulmonary resuscitation and defibrillation by pediatric residents during simulated cardiopulmonary arrests. Resuscitation. 2009;80(7):819-25. PMID: 19423210; 10.1016/j.resuscitation.2009.03.020. 19423210

[22] 22 Sawyer T, Eppich W, Brett-Fleegler M, Grant V, Cheng A. More than one way to debrief: A critical review of healthcare simulation debriefing methods. Simul Healthc. 2016;11(3):209-17. PMID: 27254527; 10.1097/sih.0000000000000148. 27254527

[23] 23 Van Heukelom JN, Begaz T, Treat R. Comparison of postsimulation debriefing versus in-simulation debriefing in medical simulation. Simul Healthc. 2010;5(2):91-7. PMID: 20661008; 10.1097/sih.0b013e3181be0d17. 20661008

[24] 24 Brown KM, Mudd SS, Hunt EA, et al. A multi-institutional simulation boot camp for pediatric cardiac critical care nurse practitioners. Pediatr Crit Care Med. 2018;19(6):564-71. PMID: 29533354; 10.1097/pcc.0000000000001532. 29533354

[25] 25 Cheng A, Nadkarni VM, Mancini MB, et al. Resuscitation education science: educational strategies to improve outcomes from cardiac arrest: a scientific statement from the American Heart Association. Circulation. 2018;138(6):e82-122. PMID: 29930020; 10.1161/cir.0000000000000583. 29930020

[26] 26 Topjian AA, Raymond TT, Atkins D, et al. Part 4: Pediatric basic and advanced life support: American Heart Association Guidelines for Cardiopulmonary Resuscitation and Emergency Cardiovascular Care. Circulation. 2020;142(16 suppl 2):s469-523. PMID: 33081526; 10.1161/cir.0000000000000901. 33081526

[27] 27 Brasil. Conselho Nacional de Saúde. Resolução n° 466, de 12 de dezembro de 2012. Dispõe sobre assegurar os direitos e deveres que dizem respeito aos participantes da pesquisa, à comunidade científica e ao Estado e atualiza a resolução 196. DOU. 2013;(12 seção 1):59.

[28] 28 Brasil. Lei n° 13.709, de 14 de agosto de 2018. Dispõe sobre a proteção de dados pessoais e altera a Lei n° 12.965, de 23 de abril de 2014 (Marco Civil da Internet). Diário oficial da União. 2018;155(157 seção 1):59-64.

[29] 29 Ericsson KA. Acquisition and maintenance of medical expertise: a perspective from the expert-performance approach with deliberate practice. Acad Med. 2015;90(11):1471-86. PMID: 26375267; 10.1097/acm.0000000000000939. 26375267

[30] 30 Hunt EA, Duval-Arnould JM, Chime NO, et al. Integration of in-hospital cardiac arrest contextual curriculum into a basic life support course: A randomized, controlled simulation study. Resuscitation. 2017;114:127-32. PMID: 28323084; 10.1016/j.resuscitation.2017.03.014. 28323084

[31] 31 Gupta R, Fitzgibbons C, Ramsay C, et al. Development and pilot of an interprofessional pediatric resuscitation program for non-acute care inpatient providers. Med Educ Online. 2019;24(1):1581521. PMID: 30811308; 10.1080/10872981.2019.1581521. PMC6394294 30811308

[32] 32 Magee MJ, Farkouh-Karoleski C, Rosen TS. Improvement of immediate performance in neonatal resuscitation through rapid cycle deliberate practice training. J Grad Med Educ. 2018;10(2):192-7. PMID: 29686759; 10.4300/jgme-d-17-00467.1. PMC5901799 29686759

[33] 33 Hunt EA, Duval-Arnould JM, Nelson-McMillan KL, et al. Pediatric resident resuscitation skills improve after “Rapid Cycle Deliberate Practice” training. Resuscitation. 2014;85(7):945-51. PMID: 24607871; 10.1016/j.resuscitation.2014.02.025. 24607871

[34] 34 Lemke DS, Fielder EK, Hsu DC, Doughty CB. Improved team performance during pediatric resuscitations after rapid cycle deliberate practice compared with traditional debriefing. Pediatr Emerg Care. 2019;35(7):480-6. PMID: 27741071; 10.1097/pec.0000000000000940. 27741071

[35] 35 Kosoko AA, Glomb NW, Laba B, et al. Evaluating a Novel Simulation Course for Prehospital Provider Resuscitation Training in Botswana. West J Emerg Med. 2019;20(5):731-9. PMID: 31539330; 10.5811/westjem.2019.6.41639. PMC6754192 31539330

[36] 36 Chancey RJ, Sampayo EM, Lemke DS, Doughty CB. Learners' experiences during rapid cycle deliberate practice simulations. Simul Healthc. 2019;14(1):18-28. PMID: 30216277; 10.1097/sih.0000000000000324. 30216277

